# Effect of Polypropylene Fibre Addition on Properties of Geopolymers Made by 3D Printing for Digital Construction

**DOI:** 10.3390/ma11122352

**Published:** 2018-11-22

**Authors:** Behzad Nematollahi, Praful Vijay, Jay Sanjayan, Ali Nazari, Ming Xia, Venkatesh Naidu Nerella, Viktor Mechtcherine

**Affiliations:** 1 Centre for Sustainable Infrastructure, Faculty of Science, Engineering and Technology, Swinburne University of Technology, 3122 Melbourne, Australia; jsanjayan@swin.edu.au (J.S.); alinazari@swin.edu.au (A.N.); mxia@swin.edu.au (M.X.); 2 Institute of Construction Materials, Faculty of Civil Engineering, TU Dresden, 01062 Dresden, Germany; praful.vijay@tu-dresden.de (P.V.); venkatesh_naidu.nerella@tu-dresden.de (V.N.N.); 3 China University of Mining and Technology, Xuzhou 221116, China

**Keywords:** geopolymer, additive manufacturing, extrusion-based 3D-printing, fibre reinforcement, mechanical properties, properties in fresh state, digital construction

## Abstract

This paper investigates the effect of polypropylene (PP) fibres on the fresh and hardened properties of 3D-printed fibre-reinforced geopolymer mortars. Different percentages of PP fibres ranging between 0.25% and 1.00% by volume were added to an optimised geopolymer mixture. All samples showed reasonable workability and extrudability. In addition, shape-retention ability in the fresh state was investigated as a major requirement for 3D-printing. The compressive strength of the printed specimens was tested in the hardened state in three loading directions, viz. longitudinal, perpendicular, and lateral. The flexural strength of samples was also tested in the longitudinal and lateral directions. In addition, the interlayer bond strength was investigated. Fibre addition seems to influence compressive strengths positively only when the loading is perpendicular to the interface plane. This is due to the preferential fibre alignment parallel to the direction of extrusion. The addition of fibre significantly enhanced the flexural performance of the printed samples. The use of fibre dosages of 0.75 and 1.00 vol % caused deflection-hardening behaviour of the 3D-printed geopolymers and, hence, a significantly higher fracture energy in comparison to specimens without fibre or with lower fibre content. However, an increase in the fibre volume caused some minor reduction in interlayer bond strength. With respect to properties in the fresh state, higher fibre volumes caused better shape-retention ability in the printed samples. The results indicate the possibility of printing fibre-reinforced geopolymers which meet all the necessary properties in both the fresh and hardened states.

## 1. Introduction

3D-printing, an automated layer-by-layer production process, has been gaining significant attention in the construction industry in recent years. Although this approach has been adopted in the manufacturing industry for decades, it has been only recently introduced in the construction industry to construct free-form concrete structures digitally [1,2,3,4]. The 3D-concrete-printing (3DCP) technology can bring significant benefits to the construction industry in terms of increased customisation, reduced construction time, as well as reduced manpower and construction cost [4]. It is therefore necessary for the construction industry to understand the technology and its challenges for better utilisation in the future [3]. Numerous studies have been conducted on the related areas to expand the applicability of 3DCP in the construction industry. 

Two major techniques are emerging for 3DCP for construction applications. One is the powder-based technique which is a typical additive manufacturing process, in which a liquid binder is jetted selectively through the nozzle(s) on a layer of powder, which causes the powder particles to bind to each other. Structures with intricate shapes and fine details can be produced by this technique. The powder-based technique is an off-site process, which is appropriate for making building components with complex geometries such as panels, permanent formworks and interior structures, which can be later assembled on-site. There is a demand in the construction industry for such components, which currently can only be made with using expensive formworks based on the available construction systems. D-shape [5] and Emerging Objects [6] are two examples of 3DCP technologies which are developed based on the powder-based technique. In 2008, D-shape in collaboration with Shiro Studio succeeded to manufacture the Radiolaria pavilion with a very complex geometry measuring 3 m × 3 m × 3 m using sand as the build material and magnesium oxychloride cement (also known as Sorel cement) as the binding agent [5].

The other technique is the selective material deposition technique, also often referred to as the extrusion-based technique, in which a low slump concrete is extruded via a nozzle mounted on a gantry, crane, or robotic arm. The process could be adopted in constructing large-scale concrete structures with complex geometries. The first extrusion-based technique, known as Contour Crafting, was introduced by Khoshnevis [7]. Because of (1) the rapid construction of large-scale scaffolds and (2) feasible construction using nearly any type of low-slump concrete in conjunction with adequately chosen process parameters, the technology was already able to be implemented in a number of pilot and commercial projects. For example, an entire 400 m^2^ two-story villa was 3D printed ‘on-site’ in 45 days by the Huashang Tengda company in China [8]. In addition, the Siam Cement Group (SCG) in Thailand in collaboration with Supermachine Studio succeeded to 3D print a 3 m tall cave structure called the “Y-Box Pavilion, 21st-century Cave” [9]. Not only large-scale buildings, such as houses, could be built using this method, but this could also be achieved without any formwork. Considering that formwork cost is estimated at between 35–60% of the overall cost of concrete construction [10], the new technology is promising with respect to economic and environmental viability [11].

One of the main limitations of extrusion-based 3DCP techniques is the incorporation of conventional steel reinforcement into the 3D-printing process [12,13]. As a possible solution, conventional steel bars might be partly or completely substituted by short-fibre reinforcement, thus minimizing or rendering unnecessary requirements for steel reinforcement with regard to mastering issues of cracking due to shrinkage or temperature changes and, in some cases, achieving particular load-bearing capacity and deformability. Only a few studies have yet been conducted on utilising fibres in extrusion-based 3DCP. Le et al. [14] investigated the hardened properties of propylene (PP) fibre-reinforced fine-aggregate concrete. They used PP micro-fibres to decrease shrinkage and deformation of printed concrete in its plastic state. Hambach and Volkmer [15] reinforced ordinary Portland cement (OPC) paste using three different fibres, including glass, carbon, and basalt, and achieved high flexural and compressive strengths. Soltan and Li [16] recently demonstrated the feasibility of developing a 3D-printable polyvinyl alcohol (PVA) fibre-reinforced cementitious composite exhibiting robust strain-hardening behaviour with tensile strength and strain capacity of about 6 MPa and 4%, respectively. Hiroki et al. [17] developed 3D-printable strain-hardening cement-based composites with various contents of high-density polyethylene (HDPE) fibre and used them for digitally producing small-scale walls for subsequent testing. The specimens cut out of those walls exhibited a tensile strength of up to 5.7 MPa and a tensile strain capacity of up to 3.2%.

Another key limitation of the extrusion-based 3DCP technique is the limited range of printable concretes. Conventional OPC concrete in its current form is not suitable for this technique because of its setting characteristics. In addition, it is well established that the production of OPC is highly energy- and emissions-intensive. The manufacture of each ton of OPC involves mining about 1.5 tons of limestone and releasing into the atmosphere about 0.5 tons of carbon dioxide that have been locked below ground for millions of years as part of the limestone. The emissions due to the manufacture of OPC are the fourth largest source of carbon emissions after petroleum, coal, and natural gas and are estimated to account for 5–7% of all anthropogenic emissions [18]. Therefore, it is essential to develop concretes with alternative binders which would be suitable for extrusion-based 3DCP. Geopolymer is one of the possible sustainable substitutes for OPC. Geopolymer can be made by alkaline activation of fly ash and slag, which are industrial by-products of coal-fired power stations and iron manufacture, respectively [19]. The production of fly ash-based geopolymers emit 80% less carbon dioxide and consumes 60% less energy than the production of OPC [20,21]. Apart from the environmental advantages of geopolymers as compared to OPC, geopolymers are a highly suitable material for extrusion-based 3DCP because (1) geopolymers are highly thixotropic, i.e., while flowable under shear, they exhibit high and rapidly increasing static yield stress at rest; OPC concrete exhibits thixotropy as well, but to a much lower extent [22]; and (2) geopolymers have more flexibly adjustable setting characteristics and are capable of developing higher strengths in a short period of time [19,23], which is essential to the layer-by-layer build-up process in the extrusion-based 3DCP; and (3) geopolymers have better bond characteristics with fibre reinforcement than OPC, which is often beneficial in achieving high tensile strength and ductility [24,25].

Although some researchers have tried to utilise geopolymers as the base material for extrusion-based 3DCP, there has not been any systematic work done to optimise the mixture proportions of 3D printable geopolymers. Most of the related works have been based on trial and error to achieve a mix that is both extrudable and buildable [26,27]. To tackle this problem, the authors of this paper recently investigated the effect of various mixture parameters such as the type of activator, modulus of sodium silicate, and water-to-geopolymer-solids ratio (W/GP-solids) on the printability and mechanical properties of geopolymer mixtures [27]. The results showed that the activator types and combinations have a strong influence on the extrudability and printability of the mixture as well as the strength of 3D printed geopolymers [27]. Based on the results, an optimized 3D-printable geopolymer mixture exhibiting desirable properties was developed. As a follow-up study, this paper investigates the effects of including PP fibres with different volume percentages on the fresh and hardened properties of the optimized 3D-printable geopolymer mixture.

## 2. Materials and Experimental Procedure 

### 2.1. Raw Materials

To produce geopolymer mortars, fly ash, micron-scale silica sand, an alkaline solution composed of sodium silicate and sodium hydroxide solutions, and sodium carboxymethyl cellulose (CMC) powder were used. PP fibres were used with different volume percentages to print fibre-reinforced geopolymer mortars.

The fly ash (FA) used in this paper was sourced from the Gladstone power station in Queensland, Australia. This FA is a low-calcium (Class F) with an average particle size of 9.32 µm. The chemical composition and particle size distribution of Gladstone FA can be found in Reference [27]. 

**Figure 1:**
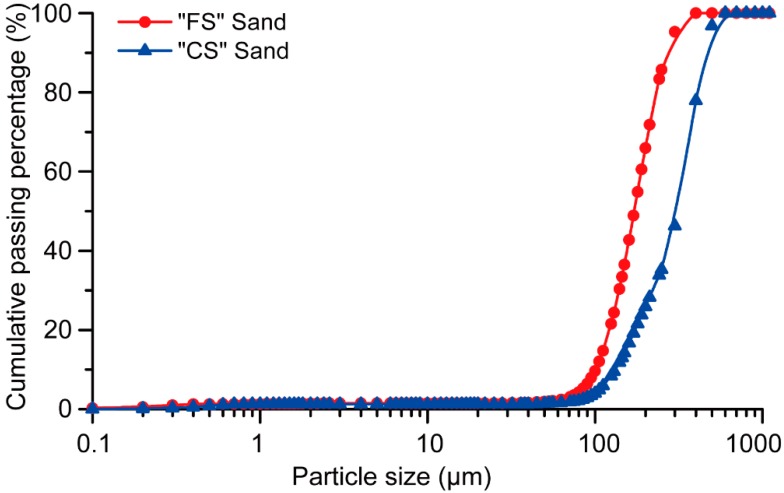
The particle size distribution of silica sands.

Fine silica sands with different particle-size distributions were used. TGS Industrial Sand Ltd. (Melbourne, Australia) sourced the finer sand (FS) with an average and maximum particle sizes of 172 μm and 271 μm, respectively. Dingo Cement Pty Ltd. (Melbourne, Australia) sourced the coarser sand (CS), where its average and maximum particle sizes were 330 μm and 465 μm, respectively. Figure 1 shows the particle size distribution of both FS and CS as obtained using a CILAS 1190 Laser Diffraction Particle Analyser (Orléans, France).

The alkaline solution used in this investigation was composed of a N Grade sodium silicate (Na_2_SiO_3_) solution and 8.0 M sodium hydroxide (NaOH) solution with a Na_2_SiO_3_/NaOH mass ratio of 2.5. The previous study by the authors [27] showed that this is the most effective activator that results in desirable fresh and hardened properties of geopolymer suitable for extrusion-based 3DCP. The N-Grade Na_2_SiO_3_ solution with 28.7 wt % SiO_2_, 8.9 wt % Na_2_O, and 62.4 wt % H_2_O was used as a part of the alkali activator. The viscosity of the sodium silicate solution at 20 °C was in the range of 100–300 cps and its unit weight was 1.38 g/cm^3^. NaOH beads with a purity of 97% supplied by Sigma-Aldrich were used to produce the sodium hydroxide solution forming a portion of the alkali activator. Tap water was added to dissolve the NaOH beads and produce the 8.0 M solution.

To adjust the viscosity of geopolymer mixes with respect to 3D-printing in terms of extrudability and buildability, sodium carboxymethyl cellulose (CMC) powder supplied by DKS Co. Ltd. (Kyoto, Japan) was used as a viscosity-modifying agent.

The high-density co-polymer PP fibres used in this paper were supplied by Redco NV, Antwerp Area, Belgium. The properties of the fibres are presented in Table 1.

### 2.2. Mixture Proportions

Geopolymer mixtures should be extrudable and buildable to be used in 3D-printing processes. The chosen criteria for a mixture to be extrudable is that it must be smoothly and steadily extruded through a nozzle to form filaments without being clogged in the path. In addition, the chosen criteria for a mixture to be buildable is that the fresh concrete needs to be “stiff” enough to retain its shape after being extruded. This stiff layer shall further support the weight of the upper layers to follow without significant deformation and collapsing. Yet it must still provide a sufficient interlayer bond between the layers [28]. Table 2 gives the mix proportions of the 3D-printable geopolymer mortars prepared in this investigation. The results of the previous studies of the authors on 3D-printable OPC-based mortar were used to determine the preliminary proportions [29,30]. Following the findings of those previous studies, the proportions of sand were kept constant in all mixtures and the binder paste was changed, i.e., OPC and water were substituted by fly ash and alkaline activator.

To understand the buildability and extrudability of geopolymer mixes, plain specimens (without fibre) were first prepared to optimize the content of each ingredient. According to the authors’ previous study [27], using an alkaline solution/FA equal to 0.380 can fulfil the requirements of both buildability and extrudability of the plain, un-reinforced geopolymer mortars. To investigate the effect of fibre reinforcement on geopolymer properties, four different fibre contents were chosen: 0.25, 0.50, 0.75, and 1.00% by volume. However, it was observed that the mixture with an alkaline solution/FA of 0.380 could not accommodate 1 vol % of fibre; it appeared dry and too stiff, which can be traced to liquid sorption of fine fibre as well as its lattice effect. Such a condition also affected fibre dispersion and formation of fibre lumps was observed. Thus, the alkaline solution/FA was increased to 0.467 to have 1 vol % of fibre in the mixture. This increase in the ratio was done with reference to the 1 vol % fibre content. Thereafter, constant alkaline solution/FA of 0.467 was also used for other fibre-reinforced mixtures, as well as for the mix without fibre (PP0). The amount of CMC powder in each mix was adjusted to achieve the desired rheological behaviour for 3DCP (visually assessed).

The mixing of geopolymer mortars was carried out in a Hobart mixer. A low-speed rotation was first selected to mix the fly ash and sands for about 1 min. Then, the alkaline solution was gradually added and the mixing was continued for about 4 min. Subsequently, the PP fibres were gradually added to ensure uniform fibre dispersion. Finally, the CMC powder was added after making sure that all ingredients were thoroughly mixed, and the mixing was continued further for about 2 min to achieve the appropriate rheology for the extrusion process (visually assessed). Visual observations of the authors confirmed that there was no sign of segregation or the bundling of fibres during mixing and preparation of all mixtures. 

### 2.3. Printing Procedure and Specimens’ Curing

**Figure 2:**
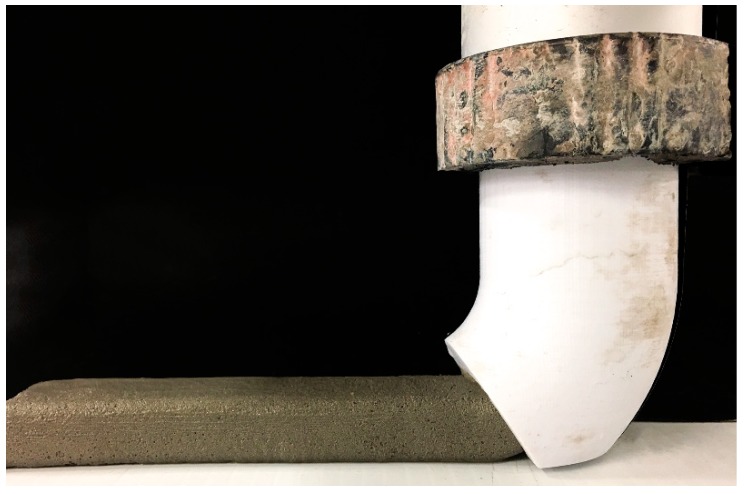
The geopolymer mortar being extruded from the nozzle.

A custom-made 3D-printing test device designed and constructed at the Swinburne University of Technology was used to simulate the extrusion-based 3DCP process. A piston-type extruder was developed for this 3D-printer to extrude the fresh material through a metallic cylinder measuring 50 mm × 600 mm (diameter × length). A 45° nozzle with a 25 mm × 15 mm opening was attached to the end of the extruder as shown in Figure 2. Additionally, also shown in Figure 2 is the geopolymer mortar being extruded through the nozzle. The fresh mixture was gradually filled into the cylinder of the extruder. A moderate external vibration was applied to the extruder to ensure complete filling and adequate compaction of the mixture inside the device. For each mixture, a single layer measuring 250 mm (L) × 25 mm (W) × 15 mm (H) was extruded by moving the extruder in the horizontal direction at a constant speed. The second layer was printed on top of the first layer with a time interval of 15 min. The same procedure was applied to all mixtures.

Heat curing was adopted in this study, for which all printed specimens were placed in a sealed container to minimize excessive moisture loss and placed in an oven at 60 °C for 24 h. At the end of the heat curing period, the specimens were removed from the oven and kept undisturbed until cooling to room temperature was completed. All specimens were stored in the laboratory at ambient temperature (23 °C ± 3 °C) until the testing day. Previous studies reported that the strength of fly ash-based geopolymer after completion of heat curing does not change significantly over time [31]. Thus, in this study, all specimens were tested one day after printing (i.e., immediately after the completion of the heat curing period and eventual cooling).

### 2.4. Testing Rheological Behaviour in the Fresh State

**Figure 3:**
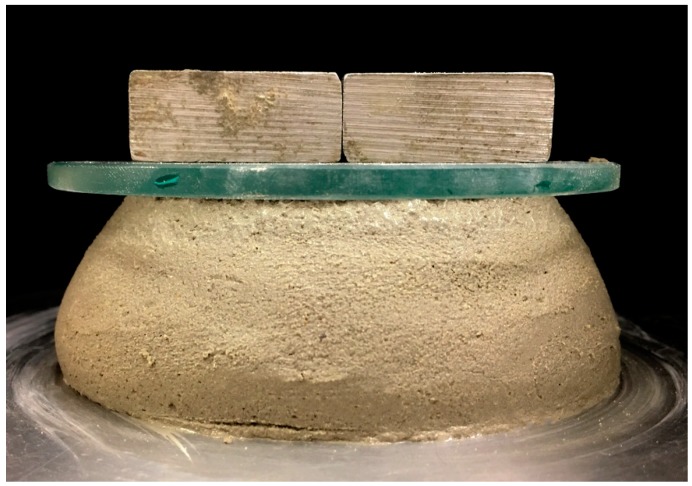
The shape-retention ability test setup.

Mini-slump tests, also known as spread-flow tests, were conducted to determine the workability of the fresh geopolymer mixtures, in accordance with ASTM C1437 [32]. To determine the capability of a printed geopolymer layer to support consecutive layers while retaining its own shape, a test called the shape-retention ability test was conducted. In this test, the fresh mixture was filled into the mini-slump cone and the cone was lifted after one minute, the material shape after lifting is referred to here as the geopolymer cone. A static load of 600 g was put on the top surface of the cone and the material was allowed to deform under the load for about 1 min. The spread of the material under the action of this static load was measured in two mutually perpendicular directions and recorded. The shape-retention ability of a mixture was characterised by the average spread diameter measured under a static load of 600 g. The lower the spread diameter, the higher the shape-retention ability of the mix. A similar test, called the plate-staking test, was used by Tay et al. [1]. Various setups were tried in applying load over the geopolymer cone. The test setup in Figure 3 was finally adopted. It involved a 100 g glass plate over the surface of the geopolymer cone and two metal weights, weighing 500 g in total, on top. The glass plate was introduced to distribute the overlying metal weight uniformly over the entire surface of the cone.

### 2.5. Testing Mechanical Properties of Hardened Mortar

**Figure 4:**
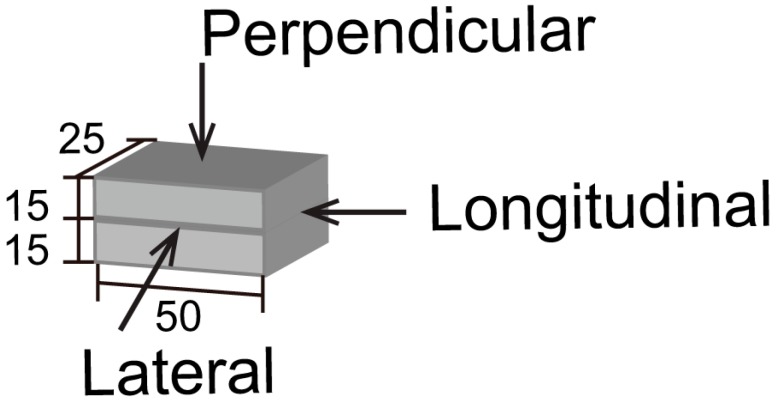
The load directions in measuring the compressive strength of the printed specimens.

To measure the compressive strength, 50 mm × 25 mm × 30 mm specimens were extracted from the printed filaments and loaded in three directions, namely the perpendicular, lateral, and longitudinal directions; see Figure 4. To reduce the end effect, the length in the longitudinal direction was chosen to be 50 mm, similar to that made by Sanjayan et al. [29]. At least 10 printed specimens were tested in each direction. All specimens were tested in uniaxial compression in a load-controlled regime at the rate of 20 MPa/min. 

To measure the flexural strength, 250 mm × 25 mm × 30 mm printed specimens were tested in two directions, namely perpendicular and lateral, using three specimens for each direction. A three-point bending test setup was used with a span of 200 mm; the tests were performed under the displacement control at the rate of 1.0 mm/min. It should be noted that in both the compression and flexural tests, the surfaces of the printed specimens loaded in different directions were ground to have a smooth and flat surface while testing. The resulting changes in the specimens’ dimensions were considered when calculating the strength values.

### 2.6. Apparent Porosity Test

The apparent porosity of the printed geopolymer mortars was measured in accordance with ASTM C20 [33]. For each mix at least 3 specimens measuring 25 mm × 25 mm × 30 mm were extracted from the printed filaments. They were placed in an oven at 105 °C for 24 h to become fully dry. The dry weight of each specimen was then measured. The specimens were then placed in water and boiled for 8 h. They were entirely covered with water and there was no contact with the heated bottom of the container. After boiling, the specimens cooled at room temperature while staying in the water. The suspended weight of each specimen was then measured. Lastly, the specimens were surface-dried using a dry cloth, and the saturated weight was measured. Using the dry weight, suspended weight and saturated weight, the apparent porosity of each mixture was calculated as per the procedure in ASTM C20 [33].

### 2.7. Interlayer Bond Test

**Figure 5:**
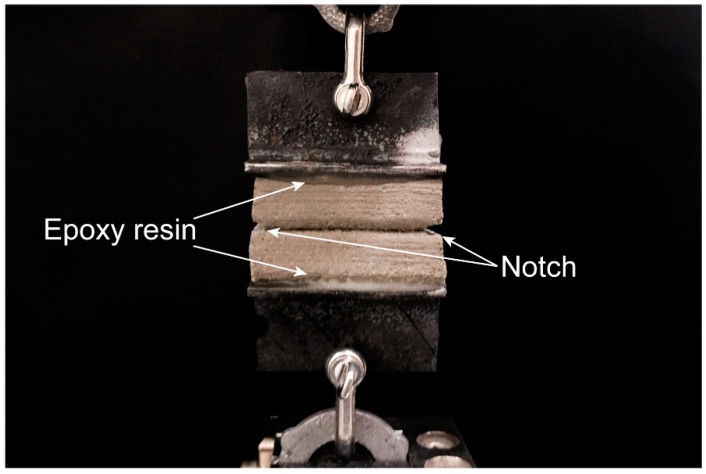
The inter-layer bond strength test of 3D-printed geopolymer mortar.

To measure inter-layer bond strength, 50 mm × 25 mm × 30 mm specimens were extracted from the printed filaments and loaded in uniaxial tension; see Figure 5. Small notches with a depth of 5 mm were cut on both edges of the layer interface to ensure the failure of the specimen at the interface. Two metallic brackets were glued to the top and bottom of each printed specimen using epoxy resin. The inter-layer bond strength test was conducted under displacement control at the rate of 1.0 mm/min. Care was taken to align the specimen in the machine to avoid any eccentricity. At least six specimens were tested for each mixture.

## 3. Results and Discussion

### 3.1. Workability and Shape-Retention Ability

It is known from the preliminary investigations that both the dosage of CMC powder and the fibre content inversely affect workability. Since a very particular rheological balance needs to be achieved to ensure both extrudability and buildability, the CMC dosage was decreased when the fibre content increased, as shown in Table 2. Table 3 presents the workability results. All mixtures exhibited similar ranges of spread diameters due to the purposeful adjustment of the CMC dosage for each mix to attain the appropriate rheology, as mentioned in Section 2.1. Despite the adjustment of the CMC dosage to balance the effect of the fibres, the PP0.75 mix exhibited the highest spread diameter. This anomalous behaviour of the PP0.75 mix requires further investigation. It should be noted that all mixtures did not exhibit any flow upon lifting of the mini-slump cone before shocks were applied to the flow table. This means that all fresh mixtures had almost zero-slump, which is desirable for the extrusion-based 3DCP process.

**Figure 6:**
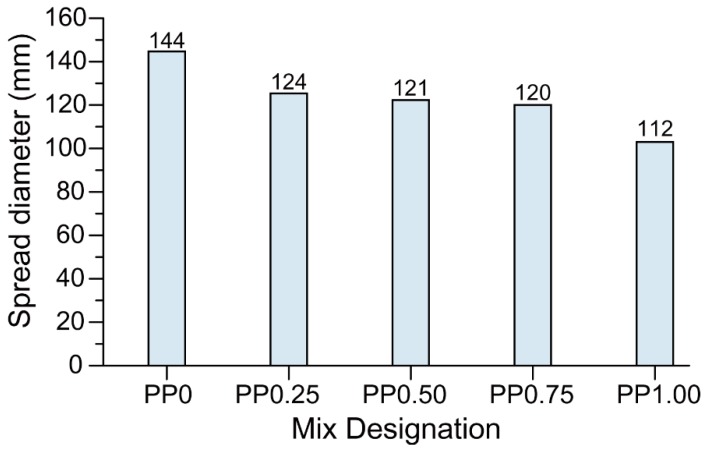
The shape-retention ability of 3D-printed geopolymer mortars in terms of the spread diameter measured under the action of the static load of 600 g.

Shape-retention ability tests revealed that better shape retention can be achieved upon increasing the fibre content as shown in Figure 6. Both fibre content and CMC dosage govern the static yield stress of the mixtures and therefore play an important role in the shape-retention ability of the fresh mixtures under external loading. As mentioned in Section 3.1, the increase in fibre content required a reduction in the CMC dosage. On the one hand, the increase of fibre dosage increased the shape-retention ability of the mixtures, on the other hand, the simultaneous decrease of the CMC content reduced the shape-retention ability. However, from the results obtained, it can be concluded that the influence of increased fibre content was more dominant than that of the reduced CMC content. Thus, the mixtures with a higher fibre content exhibited less spread diameter upon the action of static load and had a better shape-retention ability.

### 3.2. Compressive Strength and Apparent Porosity

**Figure 7:**
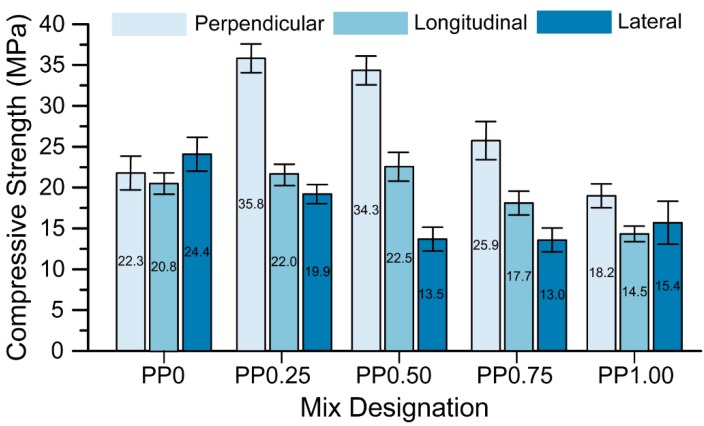
The compressive strength of printed specimens in different directions.

The compressive strength of each mix is presented in Figure 7. The compressive strength of the printed geopolymer mortars varies depending on the testing direction, thus showing an anisotropic behaviour of this material. Le et al. [14], Sanjayan et al. [29], and Panda et al. [26] also reported anisotropic behaviour of the printed OPC-based mortars and geopolymer mortars with respect to compressive strength. It should be noted that the anisotropy is more pronounced for the mixtures containing fibre as for those without fibre. While the compressive strengths of the PP0 mix in different directions were comparable, in the fibre-reinforced mixtures, the highest mean compressive strength was obtained in the perpendicular direction followed by those measured in the longitudinal and lateral directions. The reason for this trend is discussed in the following paragraphs.

**Figure 8:**
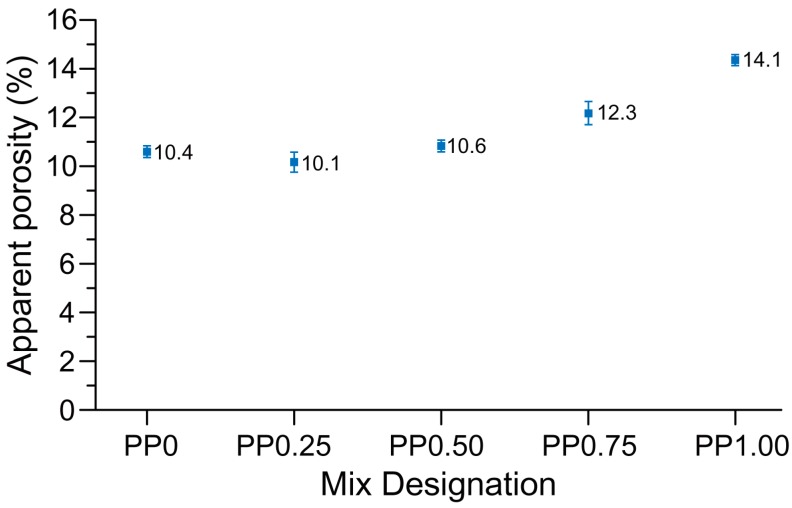
The porosity of printed fibre-reinforced specimens.

As can be seen in Figure 7, the compressive strength of the PP0 mix in the perpendicular direction was about 22 MPa. The strength value increased to about 36 MPa for the PP0.25 mix due to the addition of 0.25 vol % PP fibres. This indicates that a significant amount of fibres is aligned parallel to the extrusion direction, which helps in crack bridging upon the action of compressive force in the perpendicular direction. However, a further increase in the fibre content resulted in a reduction of the compressive strength in the perpendicular direction. This can be due to a fibre-induced increase in the entrapped air, and thereby a higher porosity of the mixture [34,35]. The porosity test results shown in Figure 8 confirm this hypothesis. The mixtures with 0.25 vol % fibre (PP0.25) and 1 vol % fibre (PP1.00) had the minimum and maximum apparent porosity, respectively. Thus, the mixture with 0.25 vol % fibre possessed the maximum compressive strength in the perpendicular direction and can be considered as the optimum fibre content with respect to the compressive strength.

According to Figure 7, the inclusion of the fibres as reinforcement does not have any significant positive effect on the compressive strength in the longitudinal direction. As mentioned above, the fibres have a preferential orientation parallel to the extrusion direction. This implies that they lie in the same plane as the loading direction when tested longitudinally. Due to this orientation, the fibres do not aid in the compressive strength. As can be seen in Figure 7, the compressive strength of PP0, PP0.25, and PP0.50 in their longitudinal directions were comparable, as these mixtures had a comparable porosity; see Figure 8. Further increases in the fibre content resulted in the reduction of the compressive strength in the longitudinal direction due to the significant increase in the porosity, as shown in Figure 8.

As mentioned earlier, the preferential direction of the fibres is parallel to the extrusion direction, and hence it can be expected from fibre reinforcement to aid to compressive strength in the lateral direction, similar to the perpendicular direction as discussed above. However, as shown in Figure 7, the compressive strength of PP0 with no fibre was about 24 MPa in the lateral direction. Upon the inclusion of the fibres and increasing the fibre content, the compressive strength in the lateral direction decreased. The low compressive strength in the lateral direction of the PP0.50, PP0.75 and PP1.00 mixtures with 0.5, 0.75, and 1% (by volume) of the fibres may be explained by the interlayer bond test results, which are discussed in Section 3.4. These three mixtures possessed a lower interlayer bond strength compared to the mixtures with 0 vol % or 0.25 vol % of fibres. The compression force in the lateral direction causes tensile and shear stresses on the interlayer. At the time of testing of these specimens, exact observations for the failure mode could not be recorded. However, assuming that the failure occurs due to the interlayer split in the specimen, these three mixtures had a lower compressive strength in the lateral direction due to the lower interlayer bond strength. Even if this explanation appears plausible to the authors, the observed anomalous behaviour (decrease in compressive strength in the lateral direction with increasing fibre content) shall be investigated more comprehensively in future research.

### 3.3. Flexural Strength

**Figure 9:**
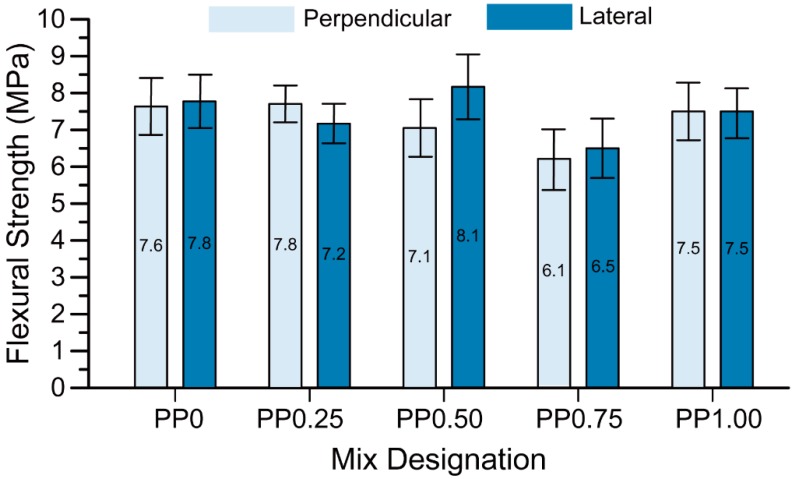
The flexural strength of printed specimens in two different directions.

The flexural strength results are presented in Figure 9. In each mix, the difference in the flexural strength values between the perpendicular and lateral directions were within the scatter of the results. Comparison between the mixtures without fibre (PP0) and with 0.25 vol % of fibres (PP0.25) showed that fibre reinforcement did not have any significant effect on the flexural strength of the mixtures. This result, unexpected at first glance, is true regardless of the testing direction. Obviously, the relatively low fibre content in combination with the relatively low elastic modulus of PP fibres does not yield a sufficient crack-bridging capacity at small deformations (relevant for flexural strength measurement) to enhance flexural strength. Moreover, the flexural strength slightly decreased with an increase in the fibre content. This increase was less pronounced in comparison to that observed for the compressive strength. While—as explained in Section 3.2—the increase in the fibre content leads to higher porosity of the mixtures and therefore to decrease in strength, the higher fibre content enhances crack bridging capacity and herewith the flexural strength, thus counteracting the negative effect of higher porosity. The detrimental effect of higher porosity on the flexural strength of PP0.50 and PP0.75 prevails over the beneficial contribution of the fibres. However, in the case of PP1.00, with the highest fibre content, the positive effect of fibre addition indeed dominates the negative effect of porosity, and so the flexural strength of this mix does not decrease despite the pronounced increase in porosity. 

**Figure 10:**
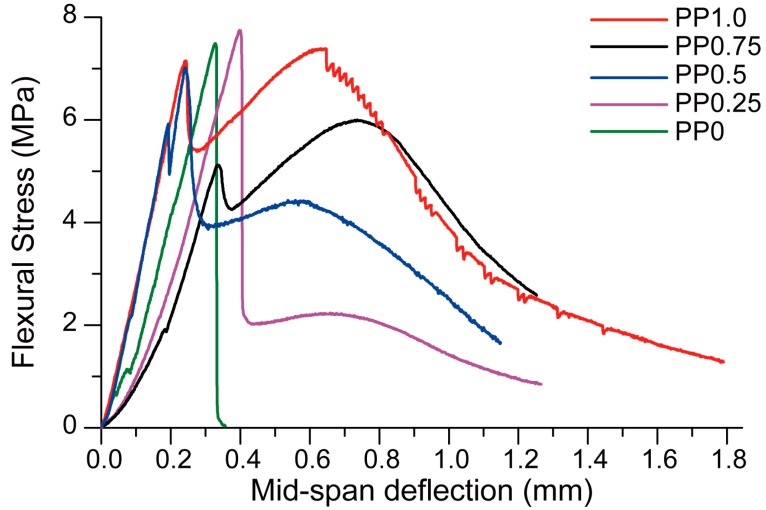
The flexural stress versus mid-span deflection curves for 3D-printed specimens subject to flexure in the perpendicular direction.

Although the addition of fibres did not contribute to the flexural strength of the printed geopolymer for the reasons explained above, it did significantly change the failure mode of the specimens. Figure 10 presents the flexural stress versus mid-span deflection behaviours of the printed specimens tested in the perpendicular direction. The mixture with no fibre (PP0) showed a brittle failure where the load dropped to zero after the specimen cracked. The inclusion of the fibres induced crack control into the printed geopolymers and changed the failure mode of the composites from brittle to ductile. The performance in terms of the ductility depended clearly on the fibre content. While the mixtures with 0.25 vol % fibre (PP.025) and 0.5 vol % fibre (PP0.50) exhibited deflection-softening behaviours, the mixtures with 1 vol % fibre (PP1.00) and 0.75 vol % fibre (PP0.75) showed deflection-hardening behaviours.

**Figure 11:**
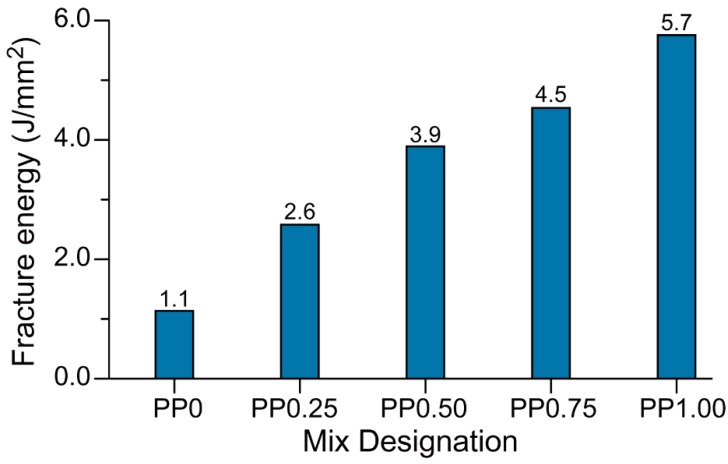
The fracture energy values of 3D-printed specimens obtained from the three-point bend tests.

Figure 11 presents fracture energy values of the printed geopolymers calculated from the area under the flexural stress versus mid-span deflection curves (Figure 10) up to 1.1-mm mid-span deflection. Observed values for fracture energy are very conclusive; they steadily increase with increasing fibre content. It is obvious that PP0.75 and PP1.00 samples have a much higher fracture energy than other samples, which is due to the deflection-hardening behaviour of these two materials. The positive effects of fibres on fracture behaviour, especially under tension and flexural loading, such as crack-bridging is well known. The pronounced proportionality of improved performance for fibre-reinforced geopolymers and fibre content confirms the earlier knowledge.

### 3.4. Interlayer Bond Strength

**Figure 12:**
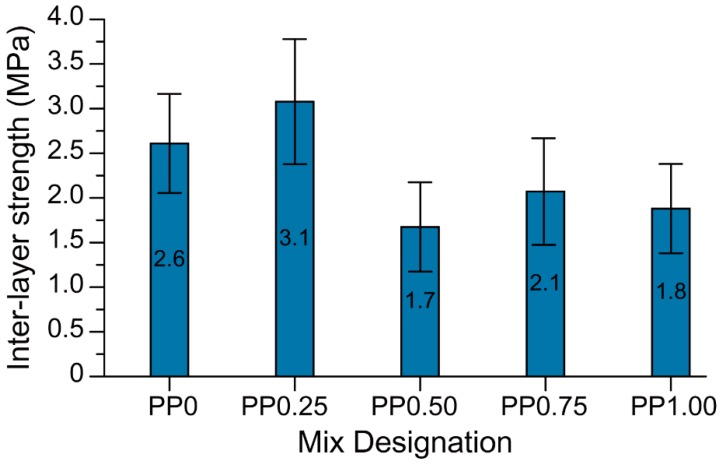
The interlayer bond strength of 3D-printed specimens.

The interlayer bond strength of each mixture under investigation is presented in Figure 12. The 3D-printed fibre-reinforced geopolymer mortars developed in this study exhibited an interlayer bond strength of 1.8 to 3.1 MPa, depending on the fibre content. The average interlayer bond strength of the PP0.25 mix containing 0.25 vol % fibre was 19% higher than that of the mix with no fibre (PP0). However, this difference was within the scatter of the results. The mixtures PP0.50, PP0.75, and PP1.00—all mixtures with a fibre content higher than 0.25 vol %—had lower interlayer bond strength than that of the PP0 and PP0.25 mixtures. In general, it can be concluded that the incorporation of fibres reduced the inter-layer bond strength of the 3D-printed geopolymer. This is consistent with the results of the previous study by the authors [36] where, regardless of the type of fibre, inclusion of fibres with a constant volume fraction reduced the inter-layer bond strength of printed geopolymer. 

It is hypothesized that the lower inter-layer bond strength of the printed fibre-reinforced geopolymer may be due to the higher stiffness of fresh fibre-reinforced mixtures owing to the inclusion of the fibres. Shape-retention ability results as shown in Figure 6 and visual observations confirmed that the mixtures containing fibres were stiffer in comparison to the mixture without fibre. The increased stiffness of the fresh mix reduced the ability of the freshly placed layers to deform and form a seamless interface. Thus, in the mixtures containing fibres, the interface of printed layers may be more porous than that of the mix without fibre, leading to a reduction in the inter-layer bond strength. As can be seen in Figure 8, the significantly higher porosity of PP0.50, PP0.75, and PP1.00 than that of PP0 supports this hypothesis. Due to the high scatter of the results, no solid conclusion can be drawn regarding the effect of the fibre content on the inter-layer bond strength of the printed geopolymer. It should be noted that the high scatter of the results was expected due to the layer-by-layer process of 3DCP and the nature of the direct tensile strength measurement and tensile bond testing of concrete repairs [37,38]. Similar high scattering of the results is reported by Le et al. [14]. It is worth noting that the inter-layer bond strength of PP0 developed in this study was significantly higher than that of the mix developed by Panda et al. [26], where the inter-layer bond strength was about 0.5 MPa for the same 15-min delay time, as compared to 2.6 MPa obtained in this study. 

With or without fibre, all printed geopolymer mortars developed in this study exhibited sufficiently high inter-layer bond strengths to prevent interfacial shear failure. This is supported by the mode of failure during flexural tests, where the flexural failures of the specimens were governed by the tensile strength of the bottom layer rather than the inter-layer shear strength.

## 4. Conclusions

This paper presented the fresh and hardened properties of 3D-printed fibre-reinforced geopolymers with varying fibre content. Two-layer printed samples were subjected to compressive and flexural testing in different loading directions to determine the hardened properties of the samples. Furthermore, the interlayer bond strength and shape-retention ability were investigated. Properties of the mixtures in the fresh state were also measured. The following results were achieved:

(1) Shape-retention ability of the material improves with increasing fibre content.
(2) The workability threshold for 3D-printing appears to be in the range of 134 mm to 158 mm mini-slump spread (after 25 drops).
(3) Fibres increase the compressive strength of the material in the perpendicular direction only. This is because of the preferential direction of fibre alignment which is parallel to the direction of the extrusion.
(4) A strong correlation between porosity and compressive strength is confirmed in 3D-printed material similar to conventionally cast concrete.
(5) Fibre reinforcement increases the ductility of the material. Increasing the fibre content leads to an increase in both the deflection capacity and fracture energy. Specimens containing 0.75 and 1.00 vol % fibres showed deflection-hardening behaviour, whereas those with 0.25 and 0.50 vol % fibres exhibited deflection-softening behaviour.
(6) Increasing the fibre volume reduced the interlayer bond strength to some extent. The drop in interlayer strength was observed when the fibre content increased beyond 0.25 vol %.

## Figures and Tables

**Table 1:** The properties of PP fibres.

Diameter (μm)	Length (mm)	Young’s Modulus (GPa)	Elongation at Rupture (%)	Density (kg/m^3^)	Nominal Strength (MPa)
11.2	6	13.2	17.6	900	880

**Table 2:** The mix proportions of 3D-printed geopolymer mortars ^1^.

Mix ID	Fly Ash	Activator	“CS” Sand	“FS” Sand	PP Fibres	CMC
PP0	1.0	0.467	1.135	0.365	-	0.040
PP0.25	1.0	0.467	1.135	0.365	0.25	0.028
PP0.5	1.0	0.467	1.135	0.365	0.50	0.023
PP0.75	1.0	0.467	1.135	0.365	0.75	0.012
PP1.00	1.0	0.467	1.135	0.365	1.00	0.004

^1^ All numbers are mass ratios of fly ash weight, except the fibre content (volume fraction).

**Table 3:** The workability results.

Mix ID	Spread Diameter ^1^ (mm)
PP0	147
PP0.25	139
PP0.5	138
PP0.75	158
PP1.00	134

^1^ After 25 times drop of the flow table.

## References

[1] Tay Y.W.D., Panda B., Paul S.C., Noor Mohamed N.A., Tan M.J., Leong K.F. (2017). 3D printing trends in building and construction industry: A review. Virtual Phys. Prototyp..

[2] Wangler T., Lloret E., Reiter L., Hack N., Gramazio F., Kohler M., Bernhard M., Dillenburger B., Buchli J., Roussel N. (2016). Digital concrete: Opportunities and challenges. RILEM Tech. Lett..

[3] Wu P., Wang J., Wang X. (2016). A critical review of the use of 3-D printing in the construction industry. Autom. Constr..

[4] Nematollahi B., Xia M., Sanjayan J. Current progress of 3D concrete printing technologies. Proceedings of the International Symposium on Automation and Robotics in Construction.

[5] Cesaretti G., Dini E., De Kestelier X., Colla V., Pambaguian L. (2014). Building components for an outpost on the lunar soil by means of a novel 3D printing technology. Acta Astronaut..

[6] Rael R., San Fratello V. Developing concrete polymer building components for 3D printing. Proceedings of the ACADIA 31st Annual Conference of the Association for Computer Aided Design in Architecture.

[7] Khoshnevis B. (2004). Automated construction by contour crafting-related robotics and information technologies. Autom. Constr..

[8] Scott C. Chinese Construction Company 3D Prints an Entire Two-Story House on-Site in 45 Days. https://3dprint.com/138664/huashang-tengda-3d-print-house/.

[9] Tess Thai Cement Maker SCG Develops an Elegant 3m-Tall 3D Printed ‘Pavilion‘ Home, 21st C. Cave. http://www.3ders.org/articles/20160427-thai-cement-maker-scg-develops-an-elegant-3m-tall-3d-printed-pavilion-home-21st-c-cave.html.

[10] Lloret E., Shahab A.R., Linus M., Flatt R.J., Gramazio F., Kohler M., Langenberg S. (2015). Complex concrete structures: Merging existing casting techniques with digital fabrication. Comput. Aided Des..

[11] De Schutter G., Lesage K., Mechtcherine V., Nerella V.N., Habert G., Agusti-Juan I. (2018). Vision of 3D printing with concrete—technical, economic and environmental potentials. Cem. Concr. Res..

[12] Mechtcherine V., Nerella V.N. (2018). Incorporating reinforcement in 3D-printing with concrete. Beton-und Stahlbetonbau.

[13] Mechtcherine V., Grafe J., Nerella V.N., Spaniol E., Hertel M., Füssel U. (2018). 3D-printed steel reinforcement for digital concrete construction—manufacture, mechanical properties and bond behaviour. Constr. Build. Mater..

[14] Le T.T., Austin S.A., Lim S., Buswell R.A., Law R., Gibb A.G.F., Thorpe T. (2012). Hardened properties of high-performance printing concrete. Cem. Concr. Res..

[15] Hambach M., Volkmer D. (2017). Properties of 3D-printed fiber-reinforced portland cement paste. Cem. Concr. Compos..

[16] Soltan D.G., Li V.C. (2018). A self-reinforced cementitious composite for building-scale 3D printing. Cem. Concr. Compos..

[17] Ogura H., Nerella V., Mechtcherine V. (2018). Developing and testing of strain-hardening cement-based composites (SHCC) in the context of 3D-printing. Materials.

[18] Huntzinger D.N., Eatmon T.D. (2009). A life-cycle assessment of portland cement manufacturing: Comparing the traditional process with alternative technologies. J. Clean. Prod..

[19] Nematollahi B., Sanjayan J., Shaikh F.U.A. (2015). Synthesis of heat and ambient cured one-part geopolymer mixes with different grades of sodium silicate. Ceram. Int..

[20] Duxson P., Provis J.L., Lukey G.C., Van Deventer J.S. (2007). The role of inorganic polymer technology in the development of ‘green concrete’. Cem. Concr. Res..

[21] Li Z., Ding Z., Zhang Y. Development of sustainable cementitious materials. Proceedings of the International Workshop on Sustainable Development and Concrete Technology.

[22] Laskar A.I., Bhattacharjee R. (2011). Rheology of fly-ash-based geopolymer concrete. ACI Mater. J..

[23] Sanjayan J. Materials technology research to structural design of geopolymer concrete. Proceedings of the 24th Australian Conference on the Mechanics of Structures and Materials.

[24] Nematollahi B., Qiu J., Yang E.-H., Sanjayan J. (2017). Microscale investigation of fiber-matrix interface properties of strain-hardening geopolymer composite. Ceram. Int..

[25] Nematollahi B., Sanjayan J., Ahmed Shaikh F.U. (2015). Tensile strain hardening behavior of PVA fiber-reinforced engineered geopolymer composite. J. Mater. Civ. Eng..

[26] Panda B., Paul S.C., Hui L.J., Tay Y.W.D., Tan M.J. (2017). Additive manufacturing of geopolymer for sustainable built environment. J. Clean. Prod..

[27] Ematollahi B., Praful V., Sanjayan J., Xia M., Nerella V., Mechtcherine V. (2018). Systematic approach to develop geopolymers for 3D concrete printing applications. Arch. Civ. Mech. Eng..

[28] Le T.T., Austin S.A., Lim S., Buswell R.A., Gibb A.G.F., Thorpe T. (2012). Mix design and fresh properties for high-performance printing concrete. Mater. Struct..

[29] Sanjayan J.G., Nematollahi B., Xia M., Marchment T. (2018). Effect of surface moisture on inter-layer strength of 3d printed concrete. Constr. Build. Mater..

[30] Marchment T., Xia M., Dodd E., Sanjayan J., Nematollahi B. Effect of delay time on the mechanical properties of extrusion-based 3D printed concrete. Proceedings of the International Symposium on Automation and Robotics in Construction.

[31] Hardjito D., Wallah S.E., Sumajouw D.M.J., Rangan A.B.V. (2004). On the development of fly ash-based geopolymer concrete. ACI Mater. J..

[32] ASTM C1437 (2007). Standard Test Method for Flow of Hydraulic Cement Mortar.

[33] ASTM C20 (2015). Standard Test Methods for Apparent Porosity, Water Absorption, Apparent Specific Gravity, and Bulk Density of Burned Refractory Brick and Shapes by Boiling Water.

[34] Nematollahi B., Sanjayan J., Qiu J., Yang E.-H. (2017). Micromechanics-based investigation of a sustainable ambient temperature cured one-part strain hardening geopolymer composite. Constr. Build. Mater..

[35] Nematollahi B., Sanjayan J., Qiu J., Yang E.-H. (2017). High ductile behavior of a polyethylene fiber-reinforced one-part geopolymer composite: A micromechanics-based investigation. Arch. Civ. Mech. Eng..

[36] Nematollahi B., Xia M., Sanjayan J., Vijay P. (2018). Effect of type of fiber on inter-layer bond and flexural strengths of extrusion-based 3D printed geopolymer. Mater. Sci. Forum.

[37] Neville A.M. (1995). Properties of Concrete.

[38] Austin S., Robins P., Pan Y. (1995). Tensile bond testing of concrete repairs. Mater. Struct..

